# Association of a Mediterranean Diet and Fruit and Vegetable Consumption with Subjective Well-Being among Adults with Overweight and Obesity

**DOI:** 10.3390/nu13041342

**Published:** 2021-04-17

**Authors:** Débora Godoy-Izquierdo, Adelaida Ogallar, Raquel Lara, Alejandra Rodríguez-Tadeo, Félix Arbinaga

**Affiliations:** 1 Departamento de Personalidad, Evaluación y Tratamiento Psicológico, Facultad de Psicología, Universidad de Granada, Campus Universitario de Cartuja, 18071 Granada, Spain; adelaidaogallar@ugr.es; 2 Grupo de Investigación Psicología de la Salud y Medicina Conductual (CTS-267), Centro de Investigación Mente, Cerebro y Comportamiento, Facultad de Psicología, Universidad de Granada, Campus Universitario de Cartuja, 18071 Granada, Spain; 3 Departamento de Psicología Social, Facultad de Psicología, Universidad de Granada, Campus Universitario de Cartuja, 18071 Granada, Spain; rlaramoreno@ugr.es; 4 Departamento de Ciencias de la Salud, Instituto de Ciencias Biomédicas, Universidad Autónoma Ciudad Juárez, Anillo Envolvente del Pronaf y Estocolmo, Ciudad Juárez 32300, Mexico; alrodrig@uacj.mx; 5 Departamento de Psicología Clínica y Experimental, Facultad de Educación, Psicología y Ciencias del Deporte, Universidad de Huelva, Campus Universitario El Carmen, 21071 Huelva, Spain; felix.arbinaga@dpsi.uhu.es

**Keywords:** healthy diet, fruits and vegetables, body image, happiness, excessive weight

## Abstract

Recent evidence suggests that among behavioral-lifestyle factors, adherence to a healthy dietary pattern such as the Mediterranean Diet (MedDiet) is linked not only to better psychological health and mental positive status but also to increased subjective well-being (SWB). Nevertheless, this association has been unexplored among individuals with excessive weight. This study explored whether adherence to the MedDiet and the intake of healthy foods such as fruits and vegetables (FV) are associated with increased happiness and life satisfaction among Spanish adults with overweight or obesity when weight, body image, and body satisfaction are also considered. A convenience sample of adult individuals with excessive weight completed self-reports on the study variables, and weight and BMI were measured by bioimpedance. No evidence of a relationship with SWB indicators was obtained for MedDiet global indicators, probably due to the low adherence to a healthy diet by these individuals. In contrast, FV intake, as a powerful indicator of healthy eating, was associated with life satisfaction when BMI and body image dimensions were considered, among which body satisfaction had a key role. Moreover, life satisfaction fully mediated the relationship between FV consumption and happiness. Our findings are expected to make a relevant contribution to knowledge on the positive correlates or protective factors for overall well-being in obesity, including dietary habits and body appreciation. Our results may inform obesity management actions focused on inclusive, positive aesthetic models and promoting a healthy lifestyle for happiness in obesity.

## 1. Introduction

Overweight (body mass index, BMI ≥ 25 kg/m^2^) and obesity (BMI ≥ 30 kg/m^2^) are recognized as major public health concerns, as they are linked to higher risks for chronic or severe somatic, mental, and social comorbidities. Body weight is associated with psychosocial ill-being in individuals with excessive weight, but research on positive states of well-being and protective factors is unfortunately scarce [1].

It has been observed that happiness is diminished among people with excess weight [2,3,4,5,6,7,8,9,10,11,12]. It is well established that subjective well-being (SWB) shows an inverse relationship with BMI. To what extent eating habits and dietary quality contribute to happiness in these individuals is unknown and constitutes the main interest in the present study.

In addition to physical functioning, health, and quality of life benefits, high-quality eating patterns such as the Mediterranean diet (MedDiet) and the consumption of some of its most healthy components (such as plant-based foods, particularly fruit and vegetables (FV) consumption) are associated—cross-sectionally, prospectively, and meta-analytically— with decreased psychological distress and mental illness, including risk of depression (e.g., [13]). With respect to positive psychological functioning and well-being, above and beyond mental disturbances, research supporting the association of the MedDiet and regular consumption of some components of a healthy diet such as high FV with indicators of positive well-being has increased in recent years (for a review, see [14,15,16] for studies on better psychological well-being, mental health, and SWB in healthy and clinical populations, and also see [17,18] for reviews on the specific effects of a healthy diet and FV consumption on indicators of SWB; all included cross-sectional and longitudinal observational studies and experimental studies).

All of these sources support the fact that a rather linear dose–response effect exists between positive indicators of mental well-being and happiness per portion of healthy foods consumed [16,17,18], with benefits with consumption rates as low as 3 servings/day and peaking with rates of 7–8 servings/day for preferably fresh but also processed (frozen, canned, cooked, etc.) products. Furthermore, a possible reverse direction of the links has been discarded [14,18]. In summary, regularly consumption close to recommended or higher amounts of healthy foods, particularly FV, results in enhanced psychological health and SWB in the short and long term, and greater improvements are to be gained when regular consumption is particularly low. Together, the findings provide strong evidence of a causal effect of healthy eating on happiness, a pattern that seems to be universal across people, times, and places and robust to the influence of confounders [17].

Unfortunately, weight status or BMI has scarcely been considered in the literature to date—it is usually taken as a controlled covariate, with some research finding no effect attributable to it (e.g., [19,20]). Some studies have included individuals with overweight and obesity in their samples (e.g., [21,22,23]), but have not analyzed possible differences. A few studies aiming to explore the influence of BMI or weight status did not support an influence for positive indicators of well-being, and contradictory findings were obtained for negative indicators; however, these studies were conducted with young adults in the normal range of weight status (e.g., [24,25,26,27]). In contrast, some findings with population-based samples (e.g., [23]) tend to support the same pattern. Thus, the impacts of overweight or obesity on the association between healthy eating (i.e., MedDiet adherence and specific healthy food consumption, such as FV) and happiness have been largely unexplored.

In the present study, we aimed to explore whether adherence to the MedDiet is associated with happiness indicators among Spanish adults with overweight and obesity, controlling for BMI, age, and sex/gender (sex/gender is used herein to emphasize the interrelation and intersectionality between the concepts, and the personal experiences, of both sex and gender). While research to date has been mostly focused on the harmful consequences of obesity for psychological ill-being, our aim was to contribute by exploring happiness and promoting psychosocial factors such as healthy diet in individuals with excessive weight. Moreover, given that negative body image and body dissatisfaction have been consistently related to decreased functioning and well-being among individuals with excessive weight [1], subjective dimensions of body perceptions, i.e., desired weight change and body satisfaction, were also considered. To our knowledge, this is the first study exploring such relationships among individuals with excess weight. We expected to support previous research conducted in this population with general samples confirming the positive relationship between the MedDiet and SWB. Moreover, we expected to support the key role of FV consumption in this relationship. We also expected to find a key role of body satisfaction in such a relationship.

## 2. Materials and Methods

### 2.1. Participants and Procedure

A total of 100 adults aged from 19 to 57 years old (average age: 42.03 ± 10.74, 60% women) residing in southern Spain voluntarily participated in the study. No differences were found for age between females and males (*t* = −1.139, *p* = 0.258) and participants with overweight and obesity (*t* = −0.362, *p* = 0.718). All participants had a BMI ≥ 25 (69% overweight: 45% overweight I, 24% overweight II; 31% obese: 22% obesity I, 9% obesity II), with no severe mental or physical disease. All of them were Mediterranean, white individuals in the average range of socioeconomic status. Social class was determined by unifying education level, job status, and income. Following the definition by the Health Determinants Taskforce of the Spanish Epidemiology Society, six categories were established, which were regrouped into three: high (classes I–II), middle (classes III–IV), and low social classes (classes V–VI) [28].

Recruitment was conducted with a convenient, nonprobabilistic procedure according to the inclusion criteria (i.e., having overweight or obesity, not suffering from severe physical and mental diseases, being 18–65 years old) in local medical settings. The sample size was estimated prior to the study using the Clinical and Translational Science Institute (University of California, San Francisco) online calculator for clinical correlational research [29]. We decided to recruit as many individuals as possible. The sample was finally composed of individuals with excess weight who sought consultation for weight and health in two collaborating primary health care centers during the approved period for recruiting participants and conducting the assessment phase (March, 2019), who met the inclusion criteria and agreed to voluntarily participate.

After inviting the individuals to voluntarily participate and after informing them about a study on well-being and health in adults with excessive weight, the anonymous nature of the data, and the research participants’ rights, written consent was obtained. Then, assessment was conducted in a medical examination room. First, sociodemographic data and self-reported weight and height information were collected in an interview format. Then, self-reported data on body perceptions, adherence to the MedDiet, and happiness were collected. The order of the questionnaires was counterbalanced to avoid order biases. Finally, objective measures of weight and height were obtained.

Approval was obtained from the ethics committee of the authors’ university (CIEB-2018-1-36). The procedures used in this study adhere to the tenets of the Declaration of Helsinki of 1975, revised in 2013.

### 2.2. Study Variables and Measures

Sociodemographic data were collected from the participants. Weight, height, and BMI were measured with a mobile anthropometer with bioelectrical impedance (Aicok Weight Scale, model CF398BLE, Beijing, China). BMI was then categorized according to international standards on nutritional status in the adult population [30], i.e., <18.5 low weight, 18.5–24.9 normal weight, 25.0–29.9 overweight, and ≥30.0 obesity.

The perceptual component of body image was explored by using silhouettes corresponding to different BMI ranges and levels of muscularity [31]. A total of 15 male or female body figures were presented to the individuals to assess their own perceived bodies (perceived body image, PBI) and ideal bodies (ideal body image, IBI) (in both cases, 1 = excessively obese, 8 = excessively thin and flaccid, 15 = excessively muscular). Based on these, we calculated the desire to change weight and body appearance by computing the PBI–IBI discrepancy (i.e., negative difference values indicate a desire for a slimmer or more muscular body; positive difference values indicate a desire for a heavier or less muscular body) [32]. In addition, body satisfaction was assessed by a single face-valid item (“How satisfied are you with your current body weight and appearance?” 1 = extremely dissatisfied, 7 = extremely satisfied) [32]. Body satisfaction is considered a key dimension in the evaluative–subjective component of body image [33].

Life satisfaction was assessed with one item from the Spanish version [34] of the 8-item positivity scale [35]. The item regarding life satisfaction (“Overall, I am satisfied with my life”, 1 = completely disagree, 5 = completely agree) was used in the present study. Single-item life satisfaction measures are common in SWB research, as they are reliable and demonstrate a substantial degree of criterion and construct validity in comparison with multiple-item life satisfaction measures (e.g., [36,37].

SWB was self-reported using the happiness scale [38]. The single-item indicator of current happiness (“How happy are you at the present, i.e., the last few days or weeks?” 0 = extremely unhappy, 10 = extremely happy) was used. Single-item indicators of happiness are usually used in national surveys and individual research, showing that measuring happiness with a single item is reliable, valid, and viable in community surveys, as well as in cross-cultural comparisons [39,40]. Thus, besides life satisfaction, SWB was also measured at a molar level [41].

Adherence to a healthy diet was assessed with the 14-point Mediterranean diet adherence screener (MEDAS) [42]. This scale assesses adherence to the MedDiet with 14 dichotomic items for different diet nutrients or food intake habits characteristic of this diet (0 = no adherence to recommendations, 1 = adherence to recommendations). The Spanish Society of Obesity [43] introduced several modifications to the MEDAS to adapt the score to a healthier diet for Spanish individuals (specifically, the recommended intake of olive oil was reduced from ≥4 to ≥2 tablespoons/day, of wine from ≥7 to ≥3 glasses/week, and of nuts from ≥3 to ≥1 serving/week) [44]. Although cutoffs have been stated for low (total score of ≤5 or ≤6), moderate (6–9 or 7–10), and high adherence (≥ 10 or ≥11) [43,45], in the present study, a MEDAS total score below 9 was considered low MedDiet adherence and a score equal to 9 or above was considered high adherence [23,46]. Moreover, several composed categories of nutrients were computed by combining the following items: vegetable + fruit intake (items 3 and 4; alternatively, vegetable + fruit + legume + nut + sofrito intake, items 3, 4, 9, 13, and 14); sugar intake (items 7 and 11); and animal-based protein and fat intake (items 5, 10, and 13). The remaining items were not considered for compound scores (e.g., the consumption of olive oil, butter, margarine, or creams was hardly categorized exclusively into one of the abovementioned categories).

### 2.3. Statistical Analyses

This was a cross-sectional correlational study. The nature and adequacy of the data were checked and parametric assumptions were confirmed before conducting the analyses. Specifically, normality and homoscedasticity were tested for the main study variables prior to analyses. Normality assumption was not meet (Kolmogorov–Smirnov test, *p* < 0.05), however skewness—ranging from −1.126 (life satisfaction) to 0.764 (diet quality global score)—and kurtosis analyses—ranging from −0.720 (ideal body image) to 1.090 (happiness)—indicated low, non-problematic departure from a normal distribution for all of the main study variables [47,48]. Moreover, homogeneity of variance was proved (e.g., values for Levene’s test for body satisfaction, life satisfaction, happiness, and diet quality global scores were 0.708, *p* = 0.402; 0.712, *p* = 0.401; 303, *p* = 0.583; and 0.589, *p* = 0.444, respectively). Thus, parametric tests were used.

Frequency analysis and descriptive statistics (n and % for categorical variables, and mean and standard deviation (M ± SD) values for continuous variables) were calculated, as well as Pearson’s *r* zero-order pairwise correlations, *t*-Student’s comparisons for independent samples, and Pearson’s χ^2^ tests for distribution comparisons. In addition, hierarchical multivariate linear regression analysis was used to examine the predictive role of healthy diet indicators on happiness endpoints, namely happiness and life satisfaction. Specifically, two multiple regression models were built for each well-being variable considered as a criterion. The first included the global score on the MEDAS and the second included each of its 14 items, as well as composite scores. In each model, BMI (as a continuous variable), desired weight change (i.e., PBI–IBI; we selected this variable instead of body image perceptions (i.e., PBI and IBI) as a better reflection of body valoration), and body satisfaction were also introduced to test their influences. All models included age and sex/gender as potential confounders. In each of the analyses, demographic variables were entered in step 1; BMI, desired weight change, and body satisfaction were entered in step 2; and the indicators of a healthy diet were entered in step 3. In all regression models, non-standardized (*b*) and standardized beta (*β*) regression coefficients (the latter as a measure of the effect sizes associated with the former regression coefficients) were calculated.

Indirect mediation analyses were conducted to explore in depth the relationships indicated by the abovementioned analyses. Specifically, both simple and multiple mediation with mediators operating in serial were tested with Hayes’ PROCESS SPSS (Statistical Package for the Social Sciences, BMI^®^) macros (models 4 and 6, respectively) [49]. A 5000-sample, bias-adjusted bootstrap procedure was performed to generate upper and lower 95% confidence intervals for indirect effect coefficients. Covariates (age, sex/gender, and BMI) did not affect the results and were removed from the final models for simplicity.

The significance level for all analyses was set at *p* < 0.05. Statistical analyses for the current study were conducted using SPSS 25.0 (SPSS Inc., Chicago, IL, USA, 2017).

## 3. Results

Table 1 shows the descriptive statistics and correlation results for BMI, body perceptions, desired body change, body satisfaction, healthy diet, and SWB indicators (happiness and life satisfaction).

Table 2 shows the frequencies of positive scores for each of the items in the MEDAS, the mean total score over 14, and the percentage of individuals in each of the adherence categories. Notably, only two in ten participants were considered to adhere to the healthy eating pattern of the MedDiet. A total of six out of the 14 items of the questionnaire were positively scored by more than half of the sample; these were mostly related to the use of olive oil as the main culinary fat (items 1, 2, and 14; almost all of the participants) and the consumption of nuts (9 in ten of the participants), legumes (2/3 of the participants), and seafood (1/2 of participants). In contrast, approximately one-third of the participants ate at least two servings of vegetables or three fruit units per day, and the majority of the sample (7–8 of ten) reported an intake above the recommendations in terms of red meat, fats, and sugar-added drinks and foods. Moreover, BMI and the total score on the MEDAS were inversely correlated (r = −0.34, *p* < 0.01). Comparisons were conducted between individuals with overweight and obesity in terms of diet quality (see Table 3). Several diet components discriminated between weight categories, including the daily consumption of fruit; as with fruit intake, daily consumption of vegetables was higher in overweight compared to individuals with obesity, however this difference was not significant. For the remaining diet components, non-significant differences were found (*p* > 0.05). In addition, there was a significantly different distribution of adherence to MedDiet patterns by BMI category (see Table 3).

We used hierarchical multiple regression to examine the effects of adherence to the MedDiet eating pattern along with BMI, desired weight change, and body satisfaction on SWB indicators while controlling for confounders. When the MEDAS total score, adherence category, or each of the MEDAS items was introduced as a predictor, only body satisfaction emerged as a significant predictor (for happiness, βs ranged from 0.43 to 0.46, *p* = 0.000; for life satisfaction, a constant β of 0.24 was obtained, *p* < 0.05; detailed results available upon request).

In contrast, the combination of nutrients emerged as a significant predictor. Table 4 shows the main findings. For the regression model involving happiness (upper panel), demographic variables accounted for 1% of the variance in step 1, and neither sex/gender nor age significantly contributed to the explained variance (correlation R^2^ = 0.01, F = 0.490, *p* = 0.614); in the second step, age, sex/gender, BMI, and PBI–IBI were not significant independent predictors, and only body satisfaction emerged as a significant predictor (β = 0.46, *p* = 0.000), accounting for 17% of the variance (correlation R^2^ = 0.17, F = 5.000, *p* = 0.000). In step 3, when all the diet-related composite scores were also introduced, only body satisfaction remained a significant predictor (β = 0.44, *p* = 0.000), accounting for 17% of the variance (correlation R^2^ = 0.17, F = 3.512, *p* = 0.001). Hence, as body satisfaction increases, independent of other factors, individuals are more likely to report being happy, and healthy diet indicators did not contribute to such a relationship.

For the regression model involving life satisfaction (Table 4, lower panel), demographic variables accounted for less than 1% of the variance in step 1, and neither sex/gender nor age significantly contributed to the explained variance (correlation R^2^ = 0.009, F = 1.442, *p* = 0.241); in the second step, age, sex/gender, BMI, and PBI–IBI were not significant independent predictors, and only body satisfaction emerged as a significant predictor (β = 0.24, *p* < 0.05), accounting for 4% of the variance (correlation R^2^ = 0.036, F = 1.737, *p* = 0.134). In step 3, when all the diet-related composite scores were introduced, FV intake (β = 0.29, *p* < 0.01) and body satisfaction (β = 0.23, *p* < 0.05) emerged as independent predictors, accounting for 9% of the variance (correlation R^2^ = 0.085, F = 2.146, *p* < 0.05). Hence, as both FV consumption and body satisfaction increase, individuals are more likely to report being satisfied with their lives independently of other factors; no other healthy diet indicator contributed to such a relationship.

When the compound variable of vegetables + fruits + legumes + nuts + sofrito was used instead of the composite relative to FV intake, similar findings were obtained. Specifically, βs were 0.24, *p* < 0.05 for body satisfaction and 0.21, *p* < 0.05 for the diet indicator for life satisfaction (β = 0.44, *p* = 0.000, and β = 0.05, *p* > 0.05, respectively, for happiness), thus indicating that the main contribution of plant-based foods was due to FV (detailed results available upon request).

**Figure 1:**
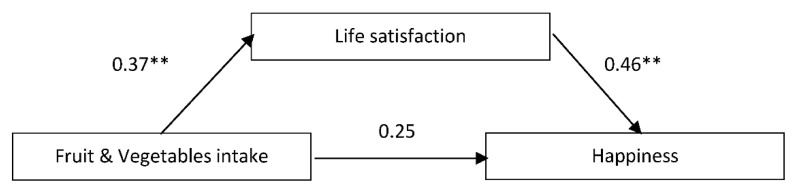
Mediational effect of life satisfaction in the relationship between FV intake and happiness. Note. Standardized coefficients for indirect and direct effects. Note: ** *p* < *0*.01.

Given the previous results, we expected to find that life satisfaction, as the cognitive component of SWB [41], was predicted by healthy eating, and in turn predicted happiness. We tested this hypothesis with a mediation analysis (PROCESS model 4), whereby FV intake was the predictor, life satisfaction was the mediator, and happiness was the predicted variable. Such a mediation effect was confirmed (see Figure 1). FV consumption explained happiness through the full mediation of life satisfaction. Table 5 displays the findings. We tentatively tested the role of body satisfaction in such a relationship. Thus, body satisfaction was proposed as mediator 1, transmitting its effects to life satisfaction as mediator 2, with both being predicted by FV intake, which in turn influenced happiness. However, no mediation effect was supported by the findings (results available upon request). This latter finding excluded body satisfaction as a possible intervening variable in the relationships between FV intake, life satisfaction, and happiness.

## 4. Discussion

The literature on SWB has largely ignored the substantial influence of diet-related factors until very recently, and research specifically with individuals with excessive weight is completely lacking. In the present study, we explored predictors of happiness, including BMI, body image perceptual–subjective and evaluative–subjective dimensions, and healthy diet in adults with overweight or obesity. This study contributes to the evidence linking the MedDiet, particularly FV consumption, to higher levels of well-being, such as positive mood, happiness, psychological well-being, and flourishing (e.g., [17,18]), underscoring the relationship between lifestyle and well-being by showing a positive association between FV consumption and SWB. With the focus shifted towards a more positive and well-being-centered perspective on eating behavior, the present study contributes to the literature by exploring such a link in individuals with excessive weight.

Our findings are parallel to other reports on SWB in Spanish adults with excess weight, which are lower than those reported for adults for all-range weight (e.g., [1,23]). In addition to shape and weight concerns, weight-related stigma, psychological distress and psychopathological symptoms, unhealthy attitudes and behaviors, the risk of chronic or fatal diseases and eating disorders, weight loss failure, and diminished quality of life, research on positive states and resources for happiness in individuals with excessive weight is scarce and the present study contributes to research in this arena.

Regarding the mean total score of the sample on the MEDAS, as expected it was lower than those reported in studies with national samples of adults from Spain [23,45,50] and other Mediterranean nations [21] that were not restricted to individuals with excessive weight. This result supports recent data suggesting that adherence to healthy eating is lower for the higher weight categories [45,46,50]. In terms of percentages of adherence, it has been reported that approximately 35% of the Spanish adult population shows high adherence (total score ≥ 9) to recommendations [45,46]; in our study, which supported expectations, lower rates were observed, supporting findings obtained with individuals with excessive weight and metabolic syndrome [51]. Our findings stress the low proportion of individuals with overweight and obesity who fully adhere to the healthy dietary patterns of the MedDiet, and moreover that individuals with excessive weight may show differences that might largely contribute to their weight. For example, when rates of adherence to each of the items of the MEDAS are compared with those reported by others for population-based samples (e.g., [23,45,46,50,52]), the participants in our study reported lower consumption of basic foods such as fruits but considerably higher intakes of solid, animal-based fats, and high-sugar foods (e.g., red meat and processed meats; butter, margarine, and creams; sugared drinks; sweets and pastries) and of healthy yet highly caloric foods (e.g., olive oil, nuts, legumes, fish, and sofrito), probably at higher quantities than that recommended. The quantity, not only the quality, of foods plays an important role in weight status [53]. It has been indicated that Spanish and other Mediterranean-based individuals are distancing from the MedDiet patterns, which is also associated with national increasing rates of excessive weight [21,23,50,54,55,56,57,58]. Recent studies suggest that the percentage of individuals who report never consuming or consuming less than 5 servings per day of FV is significantly higher in those with obesity than in subjects with normal weight [57,58]. Our findings, compared to other samples with a wider range of weight categories, may also indicate that the dietary patterns of individuals with overweight and obesity are poorer and unhealthier than those of individuals with a healthier body weight. When we compared the diet pattern of those with overweight and obesity, we also found among the latter a higher intake of highly caloric foods (e.g., high-fat meat, high-glucose foods and beverages) and lower consumption of healthy foods (e.g., fruit, legumes, white meat), and BMI was inversely correlated with adherence to the MedDiet.

It has been proposed that studying dietary patterns as a whole rather than single food components or nutrients better allow for assessment of the health effects of a diet because such patterns account for potentially cumulative effects and synergies between nutrients and foods and for possible dietary confounders; moreover, the effects of individual dietary factors may be too small to be detected. Posterior analyses of diet components can shed light on potential specific effects of some food groups or habits over others [23,53]. We approached our aims by using both types of dietary indicators. However, no effects were found when the total score for the MEDAS or the categories of adherence to the MedDiet were used, and limited findings were established for categories of foods, as we discuss in depth below. Nevertheless, correlation analyses (Table 1) demonstrated that the SWB of individuals with excessive weight is significantly or marginally related not only to the intake of healthy nutrients such as FV, but also to the consumption of unhealthy foods, such as animal-based proteins and fats, including red or processed meat products and sugar-enriched foods such as carbonated drinks. This finding has also been confirmed with other samples (e.g., [17,19,23,59]). This issue is something that future research should address.

The results of the regression models tested with each of the SWB endpoints as criterion variables and the MEDAS global score, categories of adherence, and each of the MEDAS items as independent variables did not reveal a significant relationship with any of the SWB indicators. Nevertheless, when we took into consideration the consumption of FV, we found that a higher intake of FV servings/day was related to higher life satisfaction. We could not demonstrate such an effect for happiness, with other researchers also finding discrepant results when several indicators of well-being were considered, including happiness [19,21,23,60]. However, a mediated relationship was supported, in which FV intake predicted happiness fully mediated by life satisfaction. Our findings support previous evidence, including in Spanish samples (e.g., [23]), linking FV intake to better SWB. The consumption of FV per day of at least 5 servings or units in total (MEDAS items 3 and 4) is considered one of the most relevant or common indicators of adherence to a healthy diet, since these foods are at the basis of the MedDiet pyramid [61,62,63]. Several tentative explanations for the unexpected results related to the happiness indicator and the two indicators of FV consumption include the following. It is possible that our results are due to insufficient consideration of the exact amount of FV servings per day; it has been stated that FV consumption and SWB indicators have a linear dose–response relationship [19,20,60,64,65] and that optimal intake is ≥7–8 servings per day [19,20,26], something that has also been postulated for physical and mental health outcomes [66], although others have indicated that minimal increments in consumption not reaching the “5 a day” recommendation are also associated with substantial increases in happiness [60,64], overall health, and quality of life [67]. Another possible explanation is that it has been demonstrated that raw compared to prepared FV is more associated with increments in SWB [24,27]. Future research is needed to reach more consistent findings.

The contribution of third variables is another possible reason for our findings. Regarding the association between FV intake and SWB among individuals with excess weight, our findings also confirmed the key role of body image dimensions in addition to the effects of diet-related indicators for overall well-being. Previous research has stressed the relevance of body image among individuals with excessive weight, with body dissatisfaction being higher with increasing BMI levels [68,69,70]. Most people with excess weight perceive themselves as so, even when they might not have completely accurate perceptions of their weight [31]. A considerable number of persons, particularly females, with overweight and obesity have negative body self-perceptions and are dissatisfied with their bodies and wish they were thinner. Nevertheless, we further tested whether body satisfaction could have a mediating role in the relationship between dietary quality and SWB, however could not confirm this. A previous review also discarded a mediation effect caused by better health status [17]. Research testing possible mediation effects is urgently needed.

Our findings have theoretical and clinical utility, guiding both research and treatment. Although previous evidence suggests that happiness is lower among individuals with obesity, research on the correlates of SWB in this population is warranted to increase our knowledge. This study contributes to research on happiness and its correlates in obese individuals. While weight loss has been associated with some benefits in terms of mental health and psychological well-being [71], adhering to the MedDiet could become an ecological approach to improve the affective experience and life evaluation of the population, particularly individuals who are overweight and obese, entailing benefits for and beyond happiness. Public policy programs to encourage healthy eating stress that happiness gains from healthy eating may occur much more quickly than any long-distance improvement to people’s physical health [20,22,25,26,59,60]. Nevertheless, our understanding of the complexity and heterogeneity of obesity has to improve for more successful prevention and management of obesity, and both well-being- and dieting-related factors should be monitored and targeted for their possible roles in the causation and maintenance of overweight. Furthermore, whether these associations reflect a positive cause–effect reciprocal synergism remains to be established. If so, interventions aimed at promoting a healthier lifestyle may result in increasing well-being, and vice versa.

Despite the contributions of the present study, which included considering several dimensions of body perceptions, dietary habits, and two indicators of SWB in men and women with overweight and obesity, our conclusions should be interpreted in light of some limitations. First, the sample was limited in size and constituted a nonrandom sample of individuals with excessive weight, which restricts the generalizability of our findings. Thus, the findings need to be replicated with broader and more heterogeneous samples. Second, our study relied on self-report measures that may be susceptible to various errors and biases. For instance, the use of questionnaires to assess dietary patterns may introduce an overestimation of foods considered healthy and an underestimation of foods considered unhealthy. Further research using multimodal assessment is warranted. Furthermore, it has been suggested that both hedonic and eudemonic constructs of SWB should be included to best capture associations of psychological well-being with health, quality of life, and longevity, but we restricted our analyses to indicators of hedonic well-being.

Moreover, since age, sex/gender, socioeconomic status, and lifestyle factors might have an influence on dietary behaviors, body image dimensions, and well-being indicators, but have usually been controlled for and not fully explored in the research to date on the associations between the MedDiet and SWB [17,18], future investigations should address their contributions to the link between healthy eating and happiness. Finally, due to the correlational and cross-sectional nature of the data, causal inferences cannot be stated. The utility of our findings may best be determined in future research testing the generalizability of the findings in other samples of individuals with overweight and obesity, and preferably with experimental and longitudinal analyses (e.g., whether an intervention focused on increasing adherence to the MedDiet translates into higher happiness).

In conclusion, despite the limitations, our results are pioneering and interesting. In summary, this investigation provides initial evidence that FV intake, as the basis of the MedDiet, is associated with life satisfaction in adults with overweight and obesity when BMI and body image dimensions are considered. Moreover, life satisfaction fully mediates the relationship between FV consumption and happiness. Our findings highlight the relevance of addressing both positive body image and healthy eating habits in the management of obesity and to increase happiness in this population. The current study also offers new directions for the study of well-being in obesity. Future research is required to investigate ways in which weight, body satisfaction, and dieting patterns may interact to affect functioning and well-being in individuals with excess weight and to explore how accumulated evidence may be used to inform health interventions to prevent and managing obesity in all its dimensions.

## Figures and Tables

**Table 1:** Descriptive results and correlations for the study variables and correlations between happiness and dietary components.

	M	SD	LS	HAP	FI	MI	SI	cFV	cFV+	cAN	cSU
BMI	28.76	3.57		−0.22 *							
Perceived body image (PBI)	6.17	1.96									
Ideal body image (IBI)	8.29	1.85									
PBI–IBI discrepancy	−2.12	1.79									
Body satisfaction (BS)	4.73	1.41	0.24 *	0.44 *							
MedDiet (MD, MEDAS total)	7.18	2.09		0.17							
Life satisfaction (LS)	3.88	1.09		0.31 *	0.25 *		0.19	0.26 *	0.19		
Happiness (HAP)	7.46	1.79				0.25 *	0.21 *	0.18		0.23 *	0.19

Healthy diet-related indicators: FI: fruit intake; MI: red meat intake; SI: sugar-enriched or carbonated drinks intake; cFV: composed score of FV intake; cFV+: composed score of fruit, vegetables, legumes, nuts, and sofrito intake; cAN: composed score of animal-based proteins and fats; cSU: composed score of sugar intake. Note: * *p* < 0.05; remaining: *p* < 0.10.

**Table 2:** Response frequencies to the Mediterranean diet adherence screener (MEDAS).

	Positive Scores
**MEDAS Items**	***N*** **%**
Use of olive oil as main culinary fat	91
Consumption of ≥ 2 tablespoons of olive oil per day ^a^ (including all fresh and cooked meals)	93
Consumption of ≥ 2 servings (200 g ^b^) of vegetables per day (at least 1 in salad or fresh; garnish or accompaniments, 100 g)	39
Consumption of ≥ 3 fruit units per day (including fruit juices)	35
Consumption of < 1 serving (100–150 g) of red meat or meat products (hamburger, ham, sausage, etc.) per day	20
Consumption of < 1 serving (12 g) of butter, margarine, or cream per day	17
Consumption of < 1 sweet or carbonated beverage per day	28
Consumption of ≥ 3 glasses of wine per week ^a^	16
Consumption of ≥ 3 servings (150 g) of legumes per week	66
Consumption of ≥ 3 servings (100–150 g) of fish or (4–5 units or 200 g) of shellfish per week	51
Consumption of < 3 commercial (not homemade) sweets or pastries	34
Consumption of ≥ 1 serving (30 g) of nuts per week ^a^	91
Preference for consumption of chicken, turkey, or rabbit meat (100–150 g/serving) instead of red meat, pork, or processed meat	39
Consumption of ≥ 2 times of vegetables, pasta, rice, or other dishes seasoned with “sofrito”	98
**Global MEDAS Score**	**M ± SD**
	7.18 ± 2.09
**Categories of Adherence**	***N*** **%**
Low	78
High	22

Note. ^a^ Modified by the SEEDO. ^b^ Grams are indicated for each portion, serving, or piece.

**Table 3:** Significant differences in diet indicators between participants with overweight and obesity.

MEDAS Items and Total Score	Overweight (M ± SD)	Obesity (M ± SD)	*t, p*
Consumption of ≥ 3 fruit units per day (including fruit juices)	0.42 ± 0.50	0.19 ± 0.40	2.419, 0.02
Consumption of < 1 serving (100–150 g) of red meat or meat products (hamburger, ham, sausage, etc.) per day	0.26 ± 0.44	0.06 ± 0.25	2.820, <0.01
Consumption of < 1 sweet or carbonated beverage per day	0.38 ± 0.49	0.06 ± 0.25	4.224, <0.001
Consumption of ≥ 3 servings (150 g) of legumes per week	0.72 ± 0.45	0.52 ± 0.51	1.965, 0.06 ^†^
Consumption of < 3 commercial (not homemade) sweets or pastries	0.39 ± 0.49	0.23 ± 0.43	1.713, 0.09 ^†^
Preference for consumption of chicken, turkey, or rabbit meat (100–150 g/serving) instead of red meat, pork, or processed meat	0.49 ± 0.50	0.16 ± 0.37	3.664, <0.001
**Global MEDAS Score**	7.65 ± 2.14	6.13 ± 1.54	4.024, <0.001
**Categories of adherence**	**%**	**%**	**χ^2^*, p***
Low	62.8	90.9	6.329, 0.01
High	37.2	9.1	

Note. ^†^ *p* < 0.10.

**Table 4:** Hierarchical multiple regressions of happiness (up) and life satisfaction (down) on sociodemographic, weight status, body perception and satisfaction, and MedDiet indicators (final model).

Happiness	Stand. Beta	Stand. Error	*t*	*p*
Age	−0.05	0.016	−0.474	0.636
Sex/gender	−0.06	0.381	−0.567	0.572
BMI kg/m2	−0.01	0.056	−0.042	0.967
PBI–IBI	−0.02	0.099	−0.220	0.826
Body satisfaction	0.44	0.139	4.011	0.000 **
Fruit and Vegetables (items 3 and 4)	0.13	0.240	1.289	0.201
Sugar (items 7 and 11)	0.01	0.281	0.125	0.901
Animal-based Proteins and Fats (items 5, 10, and 13)	0.06	0.219	0.565	0.574
**Life Satisfaction**	**Stand.** **Beta**	**Stand.** **Error**	***t***	***p***
Age	−0.16	0.011	−1.517	0.133
Sex/gender	0.01	0.244	0.049	0.961
BMI kg/m2	0.03	0.036	0.237	0.813
PBI–IBI	0.05	0.064	0.502	0.617
Body satisfaction	0.23	0.089	2.034	0.045 *
Fruit and Vegetables (items 3 and 4)	0.29	0.154	2.687	0.009 **
Sugar (items 7 and 11)	0.06	0.180	0.523	0.602
Animal-based Proteins and Fats (items 5, 10, and 13)	−0.13	0.140	−1.080	0.283

Note. Standard errors were robust to heteroskedasticity. Note: * *p* < *0*.05, ** *p* < *0*.01.

**Table 5:** Indirect mediational effects.

Path	Coeff.	SE	*t, p*	LLCI–ULCI
FV intake -> Life satisfaction (R^2^ = 0.07; F = 7.169, *p* = 0.009)	0.37	0.147	2.677, 0.009 **	0.096–0.643
Life satisfaction -> Happiness (R^2^ = 0.11; F = 5.854, *p* = 0.004)	0.46	0.163	2.850, 0.005 **	0.141–0.788
FV intake -> Happiness	0.25	0.231	1.084, 0.281	−0.208–0.707
Indirect effect	**Coeff.**	**Boot SE**		
	0.17	0.091		0.040–0.411

Coeff: Coefficient; SE: Standard error; LLCI: Lower limit, 95% confidence interval; ULCI: Upper limit, 95% confidence interval; ** *p* < 0.01.

## Data Availability

Data available on request due to restrictions. The data presented in this study are available on request from the corresponding author. The data are not publicly available due to privacy.

## References

[1] Godoy-Izquierdo D., González-Hernández J., Rodríguez-Tadeo A., Lara R., Ogallar A., Navarrón E., Ramirez M.J., Lopez-Mora C., Arbinaga F. (2020). Body satisfaction, weight stigma, positivity, and happiness among Spanish adults with overweight and obesity. Int. J. Environ. Res. Public Health.

[2] Blanchflower D.G., Oswald A.J., van Landeghem B. (2009). Imitative Obesity and Relative Utility. Discussion Paper No. 4010.

[3] Böckerman P., Johansson E., Saarni S.I., Saarni S.E. (2014). The negative association of obesity with subjective well-being: Is it all about health?. J. Happiness Stud..

[4] Green M.A., Strong M., Razak F., Subramanian S.V., Relton C., Bissell P. (2015). Who are the obese? A cluster analysis exploring subgroups of the obese. J. Public Health.

[5] Jansen A., Havermans R., Nederkoor C., Roefs A. (2008). Jolly fat or sad fat? Subtyping non-eating disordered overweight and obesity along an affect dimension. Appetite.

[6] Katsaiti M.S. (2012). Obesity and Happiness. Appl. Econ..

[7] Kuroki M. (2016). Life satisfaction, overweightness and obesity. Int. J. Wellbeing.

[8] Latif E. (2014). Obesity and happiness: Does gender matter?. Econ. Lett..

[9] Oswald A., Powdthavee N. (2007). Obesity, Unhappiness, and the Challenge of Affluence: Theory and Evidence.

[10] Robertson S., Davies M., Winefield H. (2015). Why weight for happiness? Correlates of BMI and SWB in Australia. Obes. Res Clin. Pract..

[11] Ul-Haq Z., Mackay D., Martin D., Smith D., Gill J., Nicholl B., Cullen B., Evans J., Roberts B., Deary I.J. (2014). Heaviness, health and happiness: A cross-sectional study of 163066 UK Biobank participants. J. Epidemiol. Community Health.

[12] Wadsworth T., Pendergast P.M. (2014). Obesity (sometimes) matters: The importance of context in the relationship between obesity and life satisfaction. J. Health Soc. Behav..

[13] Molendijk M., Molero P., Sánchez-Pedreño F.O., Van der Does W., Martínez-González M.A. (2018). Diet quality and depression risk: A systematic review and dose-response meta-analysis of prospective studies. J. Affect. Disord..

[14] Głabska D., Guzek D., Groele B., Gutkowska K. (2020). Fruit and vegetable intake and mental health in adults: A systematic review. Nutrients.

[15] Rooney C., McKinley M.C., Woodside J.V. (2013). The potential role of fruit and vegetables in aspects of psychological well-being: A review of the literature and future directions. Proc. Nutr. Soc..

[16] Tuck N.J., Farrow C., Thomas J.M. (2019). Assessing the effects of vegetable consumption on the psychological health of healthy adults: A systematic review of prospective research. Am. J. Clin. Nutr..

[17] Veenhoven R. (2019). Will healthy eating make you happier? A research synthesis using an online findings archive. Appl. Res. Qual. Life.

[18] Godoy-Izquierdo D., Lara R., Ogallar A., Mora C. (2021). Fruit and vegetable consumption and happiness in overweight and obesity: A review. Front Psychol..

[19] Blanchflower D.G., Oswald A.J., Stewart-Brown S. (2013). Is psychological well-being linked to the consumption of fruit and vegetables?. Soc. Indic. Res..

[20] Mujcic R., Oswald A.J. (2016). Evolution of well-being and happiness after increases in consumption of fruit and vegetables. Am. J. Public Health.

[21] Andrade V., Jorge R., García-Conesa M.T., Philippou E., Massaro M., Chervenkov M., Ivanova T., Maksimova V., Smilkov K., Gjorgieva Ackova D. (2020). Mediterranean diet adherence and subjective well-being in a sample of Portuguese adults. Nutrients.

[22] Ford P.A., Jaceldo-Siegl K., Lee J.W., Youngberg W., Tonstad S. (2013). Intake of Mediterranean foods associated with positive affect and low negative affect. J. Psychosom. Res..

[23] Moreno-Agostino D., Caballero F.F., Martín-María N., Tyrovolas S., López-García P., Rodríguez-Artalejo F., Haro J.M., Ayuso-Mateos J.L., Miret M. (2019). Mediterranean diet and wellbeing: Evidence from a nationwide survey. Psychol. Health.

[24] Brookie K.L., Best G.I., Conner T.S. (2018). Intake of raw fruits and vegetables is associated with better mental health than intake of processed fruits and vegetables. Front. Psychol..

[25] McMillan L., Owen L., Kras M., Scholey A. (2011). Behavioural effects of a 10-day Mediterranean diet. Results from a pilot study evaluating mood and cognitive performance. Appetite.

[26] White B.A., Horwath C.C., Conner T.S. (2013). Many apples a day keeps the blues away—Daily experience of negative and positive affect and food consumption in young adults. Brit. J. Health Psychol..

[27] Wickham S.R., Amarasekara N.A., Bartnicek A., Conner T.S. (2020). The big three health behaviors and mental health and well-being ammong young adults: A cross-sectional investigation of sleep, exercise, and diet. Front. Psychol..

[28] Domingo-Salvany A., Regidor E., Alonso J., Alvarez-Dardet C. (2020). Proposal for a social class measure. Working Group of the Spanish Society of Epidemiology and the Spanish Society of Family and Community Medicine. Atención Primaria.

[29] Hulley S.B., Cummings S.R., Browner W.S., Grady D., Newman T.B. (2013). Designing Clinical Research: An Epidemiologic Approach.

[30] World Health Organization (2017). Obesidad y Sobrepeso (Obesity and Overweight).

[31] Godoy-Izquierdo D., González J., Rodríguez A., Ramírez M., Navarrón E., Lara R., Jiménez M. (2019). «Oberexia»: The desire to be fat (ter) in adults with excess weight. Cuad. Psicol. Deporte.

[32] Godoy-Izquierdo D., González J., Lara R., Rodríguez-Tadeo A., Ramirez M.J., Navarrón E., Arbinaga F. (2020). Considering BMI, body image and desired weight change for suitable obesity management options. Span. J. Psychol..

[33] Grogan S. (2017). Body Image: Understanding Body Dissatisfaction in Men, Women, and Children.

[34] Heikamp T., Alessandri G., Laguna M., Petrovic V., Caprara M., Trommsdorff G. (2014). Cross-cultural validation of the Positivity Scale in five European countries. Pers. Indiv. Differ..

[35] Caprara G., Alessandri G., Eisenberg N., Kupfer A., Steca P., Caprara M., Abela J. (2012). The positivity Scale. Pers. Indiv. Differ..

[36] Cheung F., Lucas R.E. (2014). Assessing the validity of single-item life satisfaction measures: Results from three large samples. Qual. Life Res..

[37] Lucas R.E., Brent Donnellan M. (2012). Estimating the reliability of single-item life satisfaction measures: Results from four national panel studies. Soc. Indic. Res..

[38] Godoy-Izquierdo D., Lara R., Vázquez M.L., Araque F., Godoy J.F. (2012). Correlates of happiness among older Spanish institutionalised and non-institutionalised adults. J. Happiness Stud..

[39] Helliwell J.F., Lee M.T., Kubzanzsky L.D., VanderWeele T.J. (2021). Measuring and using happiness to support public policies. Measuring Well-Being: Interdisciplinary Perspectives from the Social Sciences and the Humanities.

[40] Pavot W., Diener E., Oishi S., Tay L. (2018). The cornerstone of research on subjective well-being: Valid assessment methodology. Handbook of Well-Being.

[41] Diener E., Suh E., Lucas R., Smith H. (1999). Subjective well-being: Three decades of progress. Psychol. Bull..

[42] Schröder H., Fitó M., Estruch R., Martínez-González M.A., Corella D., Salas-Salvadó J., Lamuela-Raventós R., Ros E., Salaverría I., Fiol M. (2011). A short screener is valid for assessing Mediterranean diet adherence among older Spanish men and women. J. Nutr..

[43] SEEDO. www.seedo.es.

[44] Estruch R., Ros E., Salas-Salvadó J., Covas M.I., Corella D., Arós F., Gómez-Gracia E., Ruiz-Gutierrez V., Fiol M., Lapetra J. (2018). Primary Prevention of Cardiovascular Disease with a Mediterranean Diet Supplemented with Extra-Virgin Olive Oil or Nuts. N. Engl. J. Med..

[45] Martínez-González M.A., García-Arellano A., Toledo E., Salas-Salvadó J., Buil-Cosiales P., Corella D., Covas M.I., Schröder H., Arós F., Gomez-Gracia E. (2012). A 14-item mediterranean diet assessment tool and obesity indexes among high-risk subjects: The predimed trial. PLoS ONE.

[46] Álvarez-Fernández C., Romero-Saldaña M., Álvarez-López A., Molina-Luque R., Molina-Recio G., Vaquero-Abellán M. (2020). Adherence to the Mediterranean diet according to occupation-based social classifications and gender. Arch. Environ. Occup. Health.

[47] Cain M.K., Zhang Z., Yuan K.H. (2017). Univariate and multivariate skewness and kurtosis for measuring nonnormality: Prevalence, influence and estimation. Behav. Res..

[48] Delacre M., Leys C., Mora Y.L., Lakens D. (2019). Taking parametric assumptions seriously: Arguments for the use of Welch’s *F*-test instead of the classical *F*-test in one-way ANOVA. Int. Rev. Soc. Psychol..

[49] Hayes A.F. (2013). Methodology in the Social Sciences. Introduction to Mediation, Moderation, and Conditional Process Analysis: A Regression-Based Approach.

[50] García-Conesa M.-T., Philippou E., Pafilas C., Massaro M., Quarta S., Andrade V., Jorge R., Chervenkov M., Ivanova T., Dimitrova D. (2020). Exploring the validity of the 14-item Mediterranean diet adherence screener (MEDAS): A cross-national study in seven European countries around the Mediterranean region. Nutrients.

[51] Alvarez-Alvarez I., Toledo E., Lecea O., Salas-Salvadó J., Corella D., Buil-Cosiales P., Zomeño M.D., Vioque J., Martinez J.A., Konieczna J. (2019). Adherence to a priori dietary indexes and baseline prevalence of cardiovascular risk factors in the PREDIMED-Plus randomised trial. Eur. J. Nutr..

[52] Lahoz C., Castillo E., Mostaza J.M., de Dios O., Salinero-Fort M.A., González-Alegre T., García-Iglesias F., Estirado E., Laguna F., Sanchez V. (2018). Relationship of the adherence to a mediterranean diet and its main components with CRP levels in the Spanish population. Nutrients.

[53] De Ridder D., Kroese F., Evers C., Adriaanse M., Gillebaart M. (2017). Healthy diet: Health impact, prevalence, correlates, and interventions. Psychol. Health.

[54] Benhammou S., Heras-González L., Ibáñez-Peinado D., Barceló C., Hamdan M., Rivas A., Mariscal-Arcas M., Olea-Serrano F., Monteagudo C. (2016). Comparison of Mediterranean diet compliance between European and non-European populations in the Mediterranean basin. Appetite.

[55] Denoth F., Scalese M., Siciliano V., di Renzo L., de Lorenzo A., Molinaro S. (2016). Clustering eating habits: Frequent consumption of different dietary patterns among the Italian general population in the association with obesity, physical activity, sociocultural characteristics and psychological factors. Eat. Weight Disord..

[56] Gomez-Donoso C., Martinez-Gonzalez M.A., Martinez J.A., Sayon-Orea C., de la Fuente-Arrillaga C., Bes-Rastrollo M. (2019). Adherence to dietary guidelines for the Spanish population and risk of overweight/obesity in the SUN cohort. PLoS ONE.

[57] Lecube A., Sánchez E., Monereo S., Medina-Gómez G., Bellido D., García-Almeida J.M., de Icaya P.M., Malagón M.M., Goday A., Tinahones F.J. (2020). Factors accounting for obesity and its perception among the adult Spanish population: Data from 1,000 computer-assisted telephone interviews. Obes. Facts.

[58] Rodríguez-Rodríguez E., Aparicio A., Aranceta-Bartrina J., Gil Á., González-Gross M., Serra-Majem L., Varela-Moreiras G., Ortega R.M. (2017). Low adherence to dietary guidelines in Spain, especially in the overweight/obese population: The ANIBES Study. J. Am. Coll. Nutr..

[59] Conner T.S., Brookie K.L., Richardson A.C., Polak M.A. (2015). On carrots and curiosity: Eating fruit and vegetables is associated with greater flourishing in daily life. Brit. J. Health Psychol..

[60] Conner T.S., Brookie K.L., Carr A.C., Mainvil L.A., Vissers M.C. (2017). Let them eat fruit! The effect of fruit and vegetable consumption on psychological well-being in young adults: A randomized controlled trial. PLoS ONE.

[61] Aune D., Giovannucci E., Boffetta P., Fadnes L.T., Keum N., Norat T., Greenwood D.C., Riboli E., Vatten L.J., Tonstad S. (2017). Fruit and vegetable intake and the risk of cardiovascular disease, total cancer and all-cause mortality—A systematic review and dose-response meta-analysis of prospective studies. Int. J. Epidemiol..

[62] Onvani S., Haghighatdoost F., Surkan P.J., Larijani B., Azadbakht L. (2017). Adherence to the Healthy Eating Index and Alternative Healthy Eating Index dietary patterns and mortality from all causes, cardiovascular disease and cancer: A meta-analysis of observational studies. J. Hum. Nutr. Diet..

[63] Schwingshackl L., Schwedhelm C., Hoffmann G., Lampousi A.-M., Knüppel S., Iqbal K., Bechthold A., Schlesinger S., Boeing H. (2017). Food groups and risk of all-cause mortality: A systematic review and meta-analysis of prospective studies. Am. J. Clin. Nutri..

[64] Ocean N., Howley P., Ensor J. (2019). Lettuce be happy: A longitudinal UK study on the relationship between fruit and vegetable consumption and well-being. Soc. Sci. Med..

[65] Warner R.M., Frue K., Morrell J.S., Carey G. (2017). Fuit and vegetable intake predicts positive affect. J. Happiness Stud..

[66] Oyebode O., Gordon-Dseagu V., Walker A., Mindell J.S. (2014). Fruit and vegetable consumption and all-cause, cancer and CVD mortality: Analysis of Health Survey for England data. J. Epidemiol. Community Health.

[67] Lalji C., Pakrashi D., Smyth R. (2018). Can eating five fruit and veg a day really keep the doctor away?. Econ. Model..

[68] Latner J., Wilson R., Cash T., Smolak L. (2011). Obesity and body image in adulthood. Body Image: A Handbook of Science, Practice, and Prevention.

[69] Silva D., Ferriani L., Viana M. (2019). Depression, anthropometric parameters, and body image in adults: A systematic review. Rev. Assoc. Méd. Bras..

[70] Weinberger N., Kersting A., Riedel-Heller S., Luck-Sikorski C. (2016). Body dissatisfaction in individuals with obesity compared to normal-weight individuals: A systematic review and meta-analysis. Obes. Facts.

[71] Jones R.A., Lawlor E.R., Birch J.M., Patel M.I., Werneck A.O., Hoare E., Griffin S.J., van Sluijs E.M., Sharp S.J., Ahern A.L. (2021). The impact of adult behavioural weight management interventions on mental health: A systematic review and meta-analysis. Obes. Rev..

